# Using Podcasts as a Learning Tool to Decrease Stigma and Increase Awareness of the Experience of Dementia

**DOI:** 10.1002/alz.089398

**Published:** 2025-01-09

**Authors:** Kathy Hickman

**Affiliations:** ^1^ Alzheimer Society of Ontario, Toronto, ON Canada

## Abstract

Dementia Dialogue is a podcast produced by the Alzheimer Society of Ontario that provides people with lived experience (people living with dementia and care partners) a way to share their stories with each other and the broader community. Listeners who have dementia and care partners gain insight and strengthen their adaptive skills. Listeners who work with people living with dementia and care partners as well as community members at large, develop an understanding of what it means to live with dementia and how these individuals can be supported, thereby increasing awareness about the experience of dementia and decreasing stigma.

A series of Learning Guides utilizing these podcasts have been created to help people with lived experience of dementia as well as others within the community, specifically professionals and volunteers supporting those with dementia and their care partners, to explore the various themes of the Dementia Journey. The Learning Guides describe key points along the dementia journey and use embedded podcast excerpts that demonstrate these themes. Excerpts are followed by reflection/discussion questions targeted to specific learner groups to aid formulation of ideas about what can be learned and how it can be applied in practice to one’s own experience. Learners can engage with the guides through independent self‐study or in group settings with a facilitator. Focus groups and surveys were conducted with people with lived experience, professionals/volunteers and educators to assess utility of the learning guides and explore the potential for development of elearning versions of the learning guides.

Delegates will learn about the podcast, learning guides and results from the testing of these.

